# A Comparison of Donor-Acceptor Pairs for Genetically Encoded FRET Sensors: Application to the Epac cAMP Sensor as an Example

**DOI:** 10.1371/journal.pone.0001916

**Published:** 2008-04-02

**Authors:** Gerard N. M. van der Krogt, Janneke Ogink, Bas Ponsioen, Kees Jalink

**Affiliations:** 1 Division of Cell Biology, The Netherlands Cancer Institute, Amsterdam, The Netherlands; 2 Division of Cellular Biochemistry, The Netherlands Cancer Institute, Amsterdam, The Netherlands; University of Oldenburg, Germany

## Abstract

We recently reported on CFP-Epac-YFP, an Epac-based single polypeptide FRET reporter to resolve cAMP levels in living cells. In this study, we compared and optimized the fluorescent protein donor/acceptor pairs for use in biosensors such as CFP-Epac-YFP. Our strategy was to prepare a wide range of constructs consisting of different donor and acceptor fluorescent proteins separated by a short linker. Constructs were expressed in HEK293 cells and tested for FRET and other relevant properties. The most promising pairs were subsequently used in an attempt to improve the FRET span of the Epac-based cAMP sensor. The results show significant albeit not perfect correlation between performance in the spacer construct and in the Epac sensor. Finally, this strategy enabled us to identify improved sensors both for detection by sensitized emission and by fluorescent lifetime imaging. The present overview should be helpful in guiding development of future FRET sensors.

## Introduction

Fluorescence Resonance Energy Transfer (FRET), the radiationless transfer of energy from a donor fluorophore to an acceptor, has become an important tool in cell biology because it allows visualization of protein-protein interactions by light microscopy. FRET is also ideally suited as the read-out for so-called ‘sensors’, i.e. genetically engineered constructs that report on the conformation or activation state of proteins. We previously reported on CFP-Epac-YFP [1], a sensor construct that consists of part of the cAMP-binding protein Epac1 sandwiched between cyan- and yellow fluorescent proteins. The construct unfolds upon binding of the second messenger cAMP to the Epac moiety and cAMP increases are thus easily followed as a drop in FRET. Whereas the cAMP-induced FRET change in CFP-Epac-YFP is already quite robust (CFP/YFP emission ratio changes by ∼30%), the recent introduction of a range of new fluorescent proteins prompted us to further optimize FRET span and other properties of the sensor by systematic variation of the donor- and acceptor fluorescent proteins.

The physical requirements for resonant energy transfer are summarized in 2 equations:
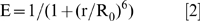
with
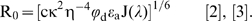
As can be seen from the inverse 6^th^ power in equation 1, transfer efficiency E depends steeply on the distance (r) between donor and acceptor, relative to the characteristic Förster radius (R_0_, the distance at which transfer is half-maximal for that particular FRET pair). R_0_ in turn depends on several factors including κ^2^ , which describes the alignment of donor- and acceptor fluorescent dipoles and J(λ), which represents the overlap of donor emission spectrum and acceptor excitation spectrum. Furthermore, donor quantum yield (ϕ_d_) and acceptor absorption coefficient (ε_a_) are important determinants of R_0_. Why these considerations are important for FRET constructs will become clear in the following.

FRET changes may be detected by several microscopical techniques [4], the most important of which are sensitized emission (SE) and fluorescence lifetime imaging (FLIM). In SE the donor is excited with light of suitable wavelength, and energy transfer is quantified from the ratio of donor and acceptor emission. Given proper correction for spectral bleedthrough and cross excitation completely quantitative results can be obtained [5]. In contrast, FLIM measurements rely on the donor signal only. In these measurements, the characteristic decay of donor fluorescence upon excitation is followed with sub-ns time resolution. FRET is then apparent as a shortening of the donor decay time, essentially as: E = 1−τ_D+A_/τ_D_, where τ_D+A_ and τ_D_ are the excited-state lifetimes of the donor in the presence and absence of the acceptor, respectively.

Recent years have seen an enormous expansion in the number of available fluorescent proteins (FPs). Almost every month new versions that differ in color, brightness or other characteristics are being added. Many of these have potential for use in FRET pairs. What makes a good FRET pair for *in vivo* sensors? Keeping with the example of CFP-Epac-YFP, even if we ignore the design considerations for the cAMP-sensing core and focus on just the FPs, the answer is already complicated. Loosely grouped, we can distinguish photophysical-, biological- and detection considerations.

### Photophysics

Obviously, for optimal FRET imaging one needs a constellation that maximizes FRET span when cAMP levels change. Thus, Förster radius of the FRET pair, brightness and dipole orientation enter the equation. Other important photophysical properties concern photostability (e.g. bleach rate and photochromism [6]), insensitivity to microenviromental conditions such as pH or ionic strength [7] and, in case of uncaging experiments, insensitivity to UV light. Finally, FPs must fold efficiently at 37°C and they must rapidly attain their final spectral properties (maturation).

### Biology

As sensors must be biologically inert, the fluorescent moieties may not influence cellular function and localization of the tagged protein. For that matter, the size of attached fluorophores must be minimal and their tendency to dimerize or aggregate excluded [8]. In general, longer excitation wavelengths are preferred over near-UV or blue excitation for reasons of phototoxicity [9] [10] [11], tissue penetration and autofluorescence. Unfortunately, many red FPs start their life as immature green protein which might complicate their use in FRET applications [12]. Obviously, this makes quick maturation an extremely important parameter.

### Detection

To complicate things even further, different detection techniques stress different qualities of the FPs. For example, low cross-excitation and high acceptor brightness (i.e., high quantum yield and high absorption) are important for SE determinations, whereas for FLIM detection these factors are less important. In fact, a high acceptor quantum yield may even be unfavorable for FLIM [13]. Conversely, SE is insensitive to multi-exponential decay of the fluorescent donor whereas FLIM analysis is severely hampered when more than one decay constant is present [14]. For another example, the photomultiplier detectors generally used in confocal imaging setups are most sensitive in the blue range of the spectrum, whereas the charge-coupled device (CCD) or avalanche photodiode (APD) detectors found in most FLIM setups favor red colors.

In all, an overwhelming amount of design considerations dazzle the beginner FRET constructionist.In this paper we describe the results of a study aimed at optimizing FRET pairs for use in biosensors like the Epac-based cAMP sensor. Our strategy was to create a wide selection of FP combinations separated by a short spacer, express them in HEK293 cells and test for FRET efficiency and other relevant properties mentioned above. Besides spectral variants, we also included dipole orientation mutants and constructs with duplicate (tandem) acceptors in our study. The most promising pairs were then cloned into the Epac FRET sensor and tested for performance as cAMP indicator in living cells. Based on the above-mentioned design considerations, we identify two of the new sensors, CFP-Epac-cp^173^Venus and GFPΔ-Epac-mRFP, as improved alternatives for SE and FLIM detection, respectively. The results further show overall good correlation between performance of FRET pairs in the linker construct and in the Epac sensor. Therefore, the present overview should be generally helpful in guiding development of new FRET sensors.

## Results

### Primary considerations and constructs included in the study

Our search for optimal FRET pairs is guided by three primary considerations.

**Figure 1:**
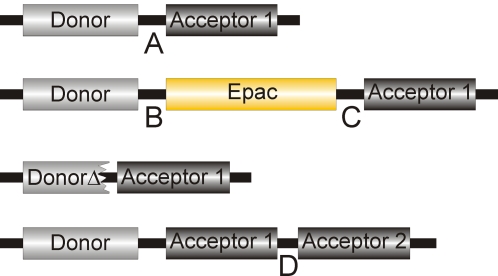
Schematic overview of the constructs used in this study. Donor and acceptor fluorophore are connected by a peptide stretch (Linker A: SGLRSRYPFASEL) or by the Epac1(ΔDEP, CD) domain [1]. Within this stretch, the amino acids PF were replaced by the Epac domain itself, leaving linkers B: SGLRSRY and C: ASEL. For truncated donor constructs (CFPΔ and GFPΔ) GITLGMDELYK was deleted from the donor FPs and SGLRS from the linker. In tandem acceptor constructs the acceptors were separated by a supplementary linker (Linker D: PNFVFLIGAAGILFVSGEL) except for tdHcRed and tdTomato which have distinctive linkers, namely NG(GA)_6_PVAT) and (GHGTGSTGSGSSGTASSEDNNMA), respectively.

(i) Optimization of FPs in the CFP-YFP part of the spectrum. The original EPAC sensor contains CFP and YFP [15]. These first-generation FPs are extremely popular for FRET, and therefore many laboratories have equipment and filters for detection in this part of the spectrum. Whereas CFP-Epac-YFP competes with the best FRET sensors in displaying robust FRET changes, we observed during extended characterization that this particular acceptor displays some reversible photochromism when excited with UV light (i.e., during cAMP uncaging experiments). Furthermore, the enhanced YFP shows some pH- and Cl^−^-dependence [16] [17] Ponsioen, unpublished observations). Therefore, the aim of this part of the study was to optimize CFP-Epac-YFP with respect to FRET span, pH resistance and UV-insensitivity. Constructs included are summarized in Figure 1–3. For details on constructs and molecular cloning the reader is referred to the Methods section.

**Figure 2 pone-0001916-g002:**
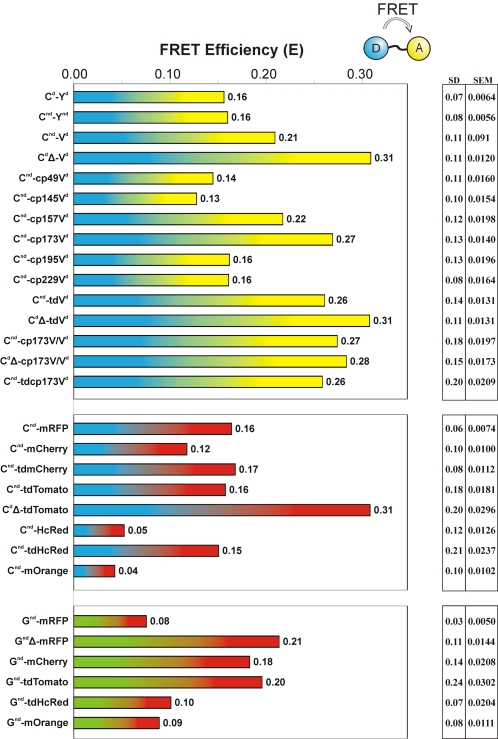
FRET in donor-linker-acceptor constructs as detected by frequency-domain FLIM. The indicated constructs were expressed in HEK293 cells and FRET efficiency E was determined as detailed in Material and Methods. Shown are mean (bars), standard deviation (SD) and standard error of the mean (SEM) of 20–400 cells. For further detail, see text.

**Figure 3:**
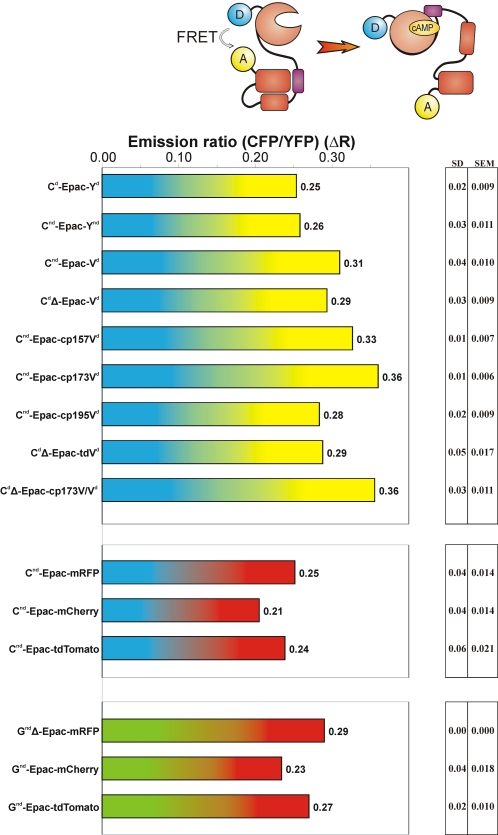
FRET span in cAMP sensors. The indicated constructs were expressed in HEK-293 cells and assayed for cAMP-induced changes in donor to acceptor ratio on a fluorescence microscope equipped with dual photometers. Donor and acceptor emission were read out simultaneously, and the baseline ratio was set to 1.0 at the onset. FRET span ΔR was determined by calculating the ratio change following addition of IBMX and Forskolin. This raises intracellular cAMP levels maximally and saturates the sensor. For further detail, see the text and Methods.

(ii) The broad emission spectrum of CFP shows considerable overlap with that of YFP. The often weak sensitized emission signals may thus be obscured in CFP leakthrough of several times its magnitude. For FRET ratiometry this is particularly unfavorable because a rise in FRET will result in opposing signals in the acceptor channel: sensitized emission of the acceptor increases, but this is masked in part by the concomitant drop in CFP leakthrough. In this part of our study, we therefore aimed to maximize FRET span by minimizing spectral overlap between donor and acceptor emission. We tested a range of red-shifted acceptors for their effectiveness in combination with the CFP donor (Figure 2–3).

(iii) Finally, we tested a series of constructs that had GFP as fluorescent donor. The rationale is fourfold: first, GFP is almost twice as bright as CFP, and second, its longer optimal excitation wavelength (489 nm versus 432 for CFP) nicely matches 488nm laser lines present in most confocal microscopes. Third, 488-nm excitation is significantly less harsh for the cells. Fourth, GFP is intrinsically better suited for FLIM measurements because its fluorescence decays mono-exponentially, as opposed to the double decay time constants observed for CFP [18] [14].

In all cases, initial experiments were performed on constructs consisting of fluorescent donors and acceptors separated by a small flexible spacer (13 amino acids; Figure 1). FRET efficiency was tested by frequency-domain FLIM on a wide-field microscope (Figure 2). Selected FP pairs were then inserted in the Epac sensor and tested for performance by FRET ratiometry (Figure 3), see Methods for details.

### CFP-YFP FRET pair analysis

#### Effect of dimerization

GFP-derivatives such as CFP and YFP have an inherent tendency to form dimers at high concentrations [19] [20]. The presence of two FPs in single-polypeptide FRET constructs multiplies this potential problem. A dimerization-disrupting mutation (A206K, [19]; here denoted as nd for non-dimerizing) was therefore introduced in both FPs in the construct (CFP^nd^-linker-YFP^nd^; Figure 2). Following expression in HEK293 cells, FRET was determined by imaging the lifetime of CFP (see Methods). Both constructs gave essentially similar FRET efficiencies (E = 0.16). Thus, dimerization does not seem to influence FRET in these constructs although conceivably the short 13-aa linker could restrict freedom of orientation in this construct, thereby masking the possible effect of the mutation.

**Figure 4:**
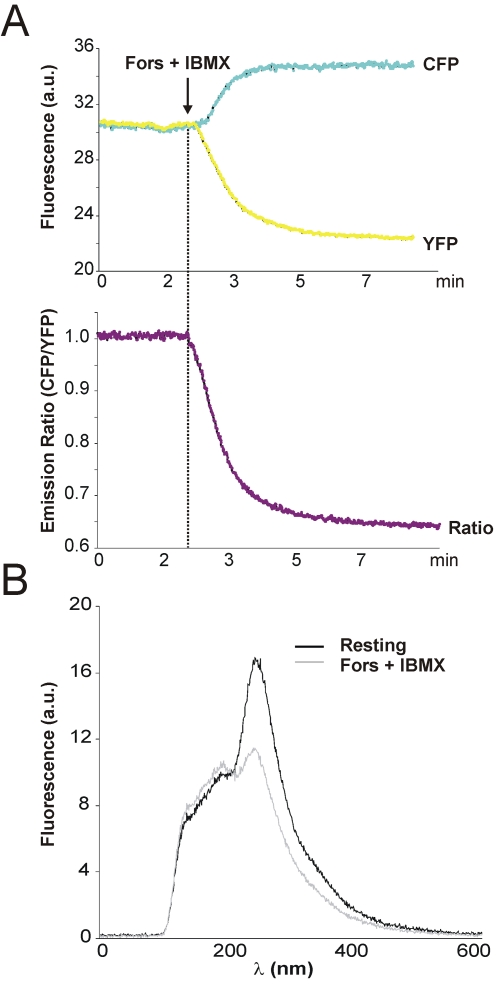
Typical FRET responses in HEK293 cell expressing CFP^nd^-Epac-cp^173^Venus to stimulation with IBMX/Forskolin. (A) CFP and YFP emission from a single HEK293 cell expressing the improved cAMP sensor were detected at 4 samples per second, following addition of IBMX and Forskolin. The YFP/CFP ratio dropped by almost 35% within minutes. Shown is a typical recording. (B) A single cell spectral fingerprint, obtained before (black) and after (grey) IBMX and Forskolin using a spectrometer. For further detail see Methods.

We next investigated the effects of nd-mutations in the Epac sensor. CFP^nd^-Epac-YFP^nd^ was prepared by inserting the cAMP-sensitive protein fragment Epac(ΔDEP, CD) [1] into the linker region. To directly compare this construct to the parental CFP-Epac(ΔDEP, CD)-YFP sensor, both were expressed in HEK293 cells and subjected to ratiometry on a dual-photometer setup (see Methods). Following recording of a baseline, cells were treated with a mix of forskolin (25 µM) and the phosphodiesterase inhibitor IBMX (100 µM) to saturate cAMP binding to EPAC [1] (Figure 4). The FRET span ΔR was then taken to be the relative drop in ratio. Remarkably, the A206K mutations had very little effect on the FRET span (ΔR = 0.26 and 0.25 for A206K (nd) and control (d), respectively; Figure 3). Thus, in both these configurations dimerization seems to be of little importance, although we anticipate that in other configurations the effects may be significant.

**Figure 5:**
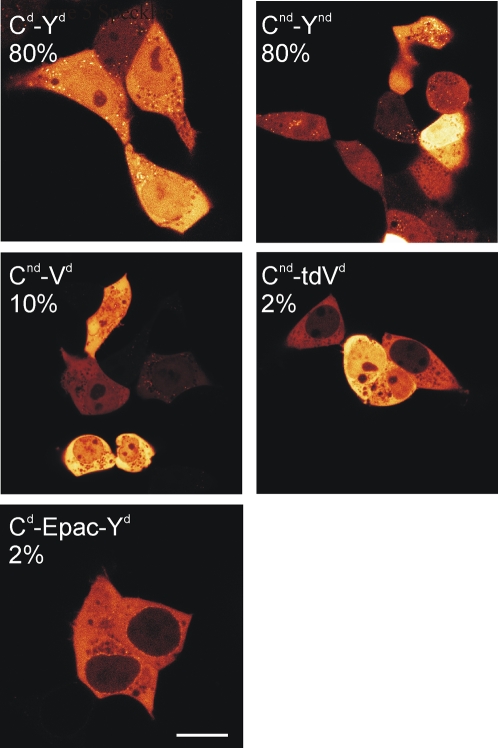
Speckle formation. HEK293 cells were transfected with indicated constructs and further cultured for 24–36 hours. Shown are confocal sections chosen to maximally visualize any speckles. Note that most speckles go unnoticed by wide-field fluorescence microscopy. Also indicated are estimated percentages of cells showing at least some speckles. Scale bar, 11 µm.

While imaging these cells by confocal microscopy, we noted that in some cell types the constructs displayed a slight tendency to form highly fluorescent aggregates (speckles) in a fraction of the cells (Figure 5). Surprisingly, the constructs containing nd mutations did not perform better in this respect. We speculate that this may be because although the dimerization constant for individual non-dimerizing FPs is significantly diminished [19], the presence of two FP moieties increases avidity of the constructs, but alternative explanations may also hold true. The insert is also an important factor determining aggregation because in the Epac(ΔDEP, CD) containing constructs speckle formation was cured almost completely.

#### Substitution of Venus for YFP

**Figure 6:**
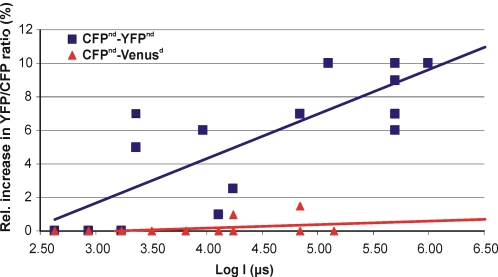
UV-induced photochromism. Change in ratio of YFP to CFP emission in CFP^nd^-linker-YFP^nd^ (squares) and CFP^nd^-linker-Venus^d^ (triangles) following exposure to UV light for the indicated times. CFP^nd^-linker-YFP^nd^ as well as free YFP (data not shown) display a dose-dependent increase in emission that maximizes at about 10%, whereas Venus and cp^173^Venus (not shown) are insensitive to UV exposure. See Methods for further detail.

From studies on YFP-expressing cells we deduced that both pH dependence and UV-induced photochromism in CFP-linker-YFP reside within the YFP moiety. We therefore replaced it by Venus [21], a variant with significantly lower pKa that therefore should be less pH-sensitive. We tested the constructs for UV-induced photochromism by subjecting cells to brief flashes of UV from a Mercury arc lamp, filtered through a Dapi filter cube (Figure 6). In YFP-containing constructs, this caused an intensity-dependent increase in YFP brightness that amounted to up to 10% in the acceptor channel. In contrast, Venus proved completely resistant to a similar UV treatment. Moreover, Venus also significantly boosted the FRET efficiency of the construct (E = 0.21) as compared to YFP (E = 0.16; Figure 2). Note however that Venus lacks the non-dimerizing mutation.

We next tested Venus in the cAMP sensor by preparing CFP-Epac-Venus. Ratiometric determination of cAMP-induced FRET changes (Figure 3) showed that Venus boosted the FRET span significantly (ΔR = 0.31, compared to ΔR = 0.25 for CFP-Epac-YFP). Thus, both in terms of UV-insensitivity and in FRET span of the sensor Venus proves a much better FRET acceptor.

#### Varying donor-acceptor distance

Systematic studies into the effect of linker length on FRET have clearly demonstrated the importance of minimizing donor-acceptor distance for FRET efficiency [22], [23]. In general, very little can be removed from the N- or C-termini of FPs without adversely influencing their performance [24]. In an attempt to minimize distance, we removed 11 C-terminal aa from CFP, as well as 5 more aa from the linker (CFPΔ; Figure 1). Indeed, removal of these 16 aa resulted in significantly improved FRET efficiency (E = 0.31, as compared to E = 0.21 for the control Venus construct; Figure 2). Note however that apart from bringing donor and acceptor closer together, this deletion may also diminish rotational freedom.

Whether the CFPΔ deletion is advantageous for any particular FRET sensor construct will of course depend on the tertiary conformation of- and rotational freedom within these chimeras. The equivalent deletion in the Epac-sensor yielded a small drop rather than an increase in FRET span (ΔR = 0.29 versus ΔR = 0.31 for the control construct CFP-Epac-Venus; Figure 3). Thus, inclusion of the CFPΔ mutation proved of no advantage for the Epac sensor.

#### Dipole alignment

FRET depends not only on spectral overlap and on donor-acceptor distance but also strongly on fluorescent dipole orientation [3]. We next set out to systematically vary the orientation of Venus to CFP by substituting the YFP acceptor with a set of orientation mutants, the so-called circularly permuted (cp) FP versions [25] [26]. In these mutants, tilting of the dipole is achieved by relocating the amino- and carboxyl termini of the acceptor fluorophore to alternative locations on the surface of the FP barrel. All 6 cpVenus versions [27] were inserted in the linker construct. FLIM analysis of cells expressing these constructs showed that only cp^173^Venus insertion resulted in a significant improvement (E = 0.27), whereas other permutations gave comparable (cp^157^) or even worse (cp^49/145/195/229^) FRET values than the precursor (E = 0.21; Figure 2).

As dipole orientation in the cAMP sensor may differ from that in the linker construct, a selection of cpVenus mutants were also tested in the Epac construct. To investigate the effect of rotation of the dipole along two axes, we tested cp195, which has its N-terminus on the same side of the barrel as wtVenus, and cp173, the N-terminus of which is on the opposing side of the barrel. Fret spans of cp^195^Venus (ΔR = 0.33) and cp^173^Venus (ΔR = 0.36) both exceeded that of the CFP-Epac-Venus construct (ΔR = 0.31; Figure 3). It was also checked that cp173 retained the favorable UV-insensitivity (Figure 6). In conclusion, both in the linker construct and in the cAMP sensor inclusion of ^cp173^Venus increases performance significantly.

#### Tandem acceptors

Finally, we studied the effect of presenting the fluorescent donors with duplicate (tandem) acceptors. This may be expected to increase FRET by raising the effective absorption of the acceptor, albeit at the cost of increased construct size. The two fluorophores in a tandem acceptor are also likely to be oriented differently, thereby easing on the requirement of donor-acceptor dipole alignment. Double acceptors have previously been used to minimize the effects of dimerization caused by FPs [28] [29]. To increase the possibility of presenting acceptors at a favorable angle, three types of tandem acceptors were made: tandem Venus, tandem cp^173^Venus, and a tandem of cp^173^Venus with Venus. As compared to the control construct CFP-linker-Venus (E = 0.21), each of the tandem receptors yielded a significantly higher FRET efficiency (range: E = 0.26–0.27; Figure 2) with CFP-linker-cp^173^Venus-Venus giving the best results. This suggests that the strategy to present the donor with acceptors at different angles may be fruitful to some degree. We therefore also combined the tandems cp^173^Venus-Venus and Venus-Venus with the effective 16aa deletion. The resulting constructs once more showed increased FRET efficiencies (E = 0.28 and 0.31, respectively), although they did not surpass the CFPΔ-linker-Venus construct (E = 0.31). In these cases, the increased construct size is thus not balanced by improved FRET efficiency. However, it is worthwhile to note that these tandem acceptor constructs displayed no tendency to form aggregates in cells (Figure 5).

Finally, we tested a subset of these FRET pairs in the Epac sensor. In these constructs, the 12aa C-terminal deletion of CFP was combined with the tandem acceptors cp^173^Venus-Venus and cp^173^Venus-cp^173^Venus. The resulting cAMP sensors showed cAMP-induced FRET changes of ΔR = 0.36 and ΔR = 0.29, respectively (Figure 3). Thus, the increased size of the construct is again not compensated by a significant increase in FRET span.

Overall, our data show significant, albeit not perfect correlation between performance of FP pairs in the linker construct and in the Epac sensor. In the linker construct, beneficial effects resulted from changing distance (the C-terminal deletion CFPΔ), orientation (cp^173^Venus), as well as from doubling the acceptor.

Importantly, incorporation of Venus in the cAMP sensor cured pH- and UV-sensitivity. Several additional alterations boosted the FRET span, including the cp^173^Venus and the tandem acceptor versions. For future experiments, the former variant (CFP-EPAC-cp^173^Venus) combines a good FRET span with small size. The CFPΔ-Epac-cp^173^Venus-Venus is the favorable candidate if speckle formation is a problem.

### CFP-RFP FRET pair analysis

As outlined before, spectral overlap of CFP and YFP complicates quantitative FRET measurements and it diminishes the FRET span of ratiometric sensors. Red-shifted acceptors could cure these drawbacks, but these are also likely to have less spectral overlap with CFP and thus less FRET. Whether this is a curse or a blessing depends on the FRET sensor at hand.

Efficient FRET from CFP to the *Discosoma* RFP (DsRed) [30] has been demonstrated before [31] [32] [33]. However, DsRed is a somewhat cumbersome acceptor because it forms tetramers and shows very slow green-to-red maturation [12]. Therefore we tested some of the newer red FPs, notably mRFP1 [28], mCherry, mOrange [8] and HcRed [34]. Also tested were tandem versions of Tomato ([8]; not tested as monomer), and of HcRed [35] and mCherry.

#### Monomeric red acceptors

In the linker construct CFP-linker-YFP, substitution of the acceptor moiety by the monomeric red acceptors mRFP1, mCherry and mOrange (Figure 2) yielded E values of 0.16, 0.12 and 0.04, respectively, as compared to E = 0.16 for the parent construct. The significant FRET in the former two constructs is somewhat surprising because these red-shifted acceptors have much less spectral overlap with the CFP donor than YFP. Note further that the “improved” successors mCherry and mOrange did not outperform their ancestor mRFP1. In fact, of these three red acceptors mOrange is the brightest and spectrally it matches CFP best [8], but it performs worst as FRET acceptor in the linker constructs. We observed that standard deviations in these experiments were relatively large (Figure 2 & 3). This may be attributed to the slow green-(em. 430–550)-to-red (em. 550–700) maturation of the red FPs [12]. Immature forms emit in the donor channel thereby biasing the fluorescence lifetimes significantly.

In the Epac sensor, we next replaced YFP with mRFP1 or mCherry, respectively. Both constructs displayed robust cAMP-induced FRET changes (ΔR = 0.24 and 0.21, respectively; Figure 3), comparable to the starting material CFP-Epac-YFP (ΔR = 0.25). Whereas donors and acceptors have better spectral separation, this did thus not materialize in improved FRET span in the ratiometric assay, and in fact the constructs were no match for sensors such as CFP-Epac-cp^173^Venus.

#### Tandem acceptors

We next prepared versions with tandem repeats of the red acceptor FPs. CFP-linker- tdTomato (E = 0.16), CFP-linker- tdmCherry (E = 0.17) and CFP-linker- tdHcRed (E = 0.15) all yielded good FRET efficiency, with the latter two slightly outperforming their respective monomeric acceptor constructs (Figure 2).

In addition, in these tandem-acceptor constructs the 16 aa deletion in the linker moiety again proved beneficial. Thus, CFPΔ-linker-tdTomato showed a FRET efficiency E = 0.31, as compared to 0.16 for CFP-linker-tdTomato. This constitutes an improvement of 0.15, and it shows that red FPs can function as very efficient acceptors for CFP.

**Figure 7:**
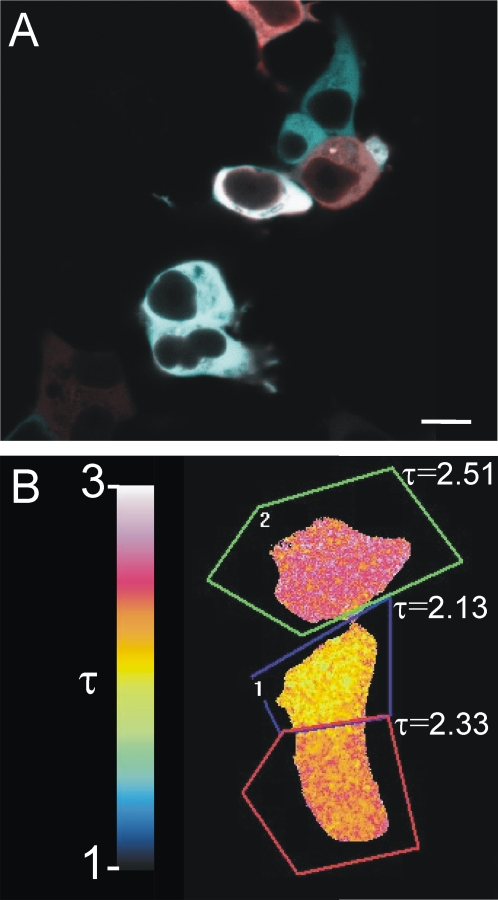
Slow green-to-red maturation of tdTomato and its effects on FRET. (A) Cells expressing CFP^nd^-EPAC-tdTomato for 24 hr display a spectrum of colors when viewed by eye using an Omega X154 triple-color (CFP-YFP-RFP) cube. For reproduction reasons, the confocal picture shows a mix of green (470–530 nm) and red (570–670) emission to closely match the image visible by eye. In contrast, CFP^nd^-EPAC-mRFP and CFP^nd^-EPAC-mCherry show a more homogeneous red color. (B) Cell-to-cell variability in maturation of CFP^nd^-linker-tdTomato causes significant deviations in the fluorescence decay times detected in the CFP channel, as measured by frequency-domain FLIM. Scale bar, 12 µm.

Finally, we tested performance of tdTomato as acceptor for CFP in the Epac sensor. CFP-Epac-tdTomato showed a robust cAMP-induced change in FRET ratio (ΔR = 0.24) but again no performance increase was observed when compared to CFP-Epac-mRFP (Figure 3). In addition, in this construct the maturation issues of tdTomato were clearly apparent. Simple visual inspection using a CFP/YFP/RFP triple filter cube plainly revealed a wide variety of maturation stages (Figure 7A). Importantly, the maturation stage of the acceptor also significantly influences E values as determined by FLIM (Figure 7B).

In conclusion, whereas these experiments show that efficient FRET from CFP to a variety of red FP acceptors is possible, the improved spectral separation did not result in superior ratiometric FRET sensors in our experiments.

### GFP-RFP FRET pair analysis

We next set out to assess performance of constructs containing GFP as FRET donor (Figure 2). As compared to CFP, the better brightness and longer excitation wavelength of GFP (489 nm versus 432 for CFP) should aid in preventing phototoxicity, and its single-exponential decay is well-suited for FLIM measurements.

Initially, we prepared GFP^nd^-linker-constructs with the red acceptors mRFP and tdHcRed (Figure 2). FRET efficiencies as detected by FLIM were E = 0.08 for GFP^nd^-linker-mRFP and E = 0.10 for GFP^nd^-linker-tdHcRed. In an attempt to boost these values, we next replicated the C-terminal deletion of the donor that had proved so effective for CFP donors. Indeed, GFP^nd^Δ-linker-mRFP showed a significantly enhanced E value of 0.21. Thus, the C-terminal deletion appears to be equally effective in GFP.

We further tested the newer variants mCherry, tdTomato and mOrange for performance with the GFP donor. GFP^nd^-linker-mCherry and GFP^nd^-linker-tdTomato showed good FRET (E = 0.18 and E = 0.20, respectively) but the value for GFP^nd^-linker-mOrange, E = 0.09, was disappointing. Thus, again, although theoretically it has a better spectral fit to the donor, mOrange did not live up to its promise in these experiments. On the other hand, the good FRET efficiency observed with mCherry is promising for future development of FLIM sensors, in particular since Time-Correlated Single Photon Counting experiments confirmed that the fluorescent decay of the GFP donor in this construct is well-described by a single exponent [36].

The most promising of these FRET pairs were subsequently tested in the Epac sensor. GFP^nd^Δ-Epac-mRFP, GFP^nd^-Epac-mCherry and GFP^nd^-Epac-tdTomato all displayed robust cAMP-induced ratio changes in HEK293 cells (ΔR = 0.29, 0.23 and 0.27, respectively; Figure 3).

Which of these constructs is recommendable as cAMP sensor in FLIM and ratiometric experiments? GFP^nd^Δ-Epac-mRFP showed the largest FRET change but mRFP photobleaches more easily than the other two [8]. For FLIM and emission-ratio experiments this should not present a problem, but acceptor bleaching would limit the useful lifespan of this construct in quantitative sensitized emission experiments [5] because for this technique separate acceptor images must be collected. The second-best construct, GFP^nd^-Epac-tdTomato, shows an almost equally large FRET span and it is very resistant to photobleaching. On the other hand, in addition to its larger size, the tdTomato moiety introduced maturation problems similar to those observed for CFP-Epac-TdTomato and we therefore do not recommend it for future experiments.

Consequently, for sensitized emission imaging GFP^nd^-Epac-mCherry is the best choice because it combines good maturation and bleaching resistance with a decent FRET span. For FLIM and simple ratiometric experiments GFP^nd^Δ-Epac-mRFP is preferred.

### Conclusions and discussion

This study aimed 1) to determine optimal donor – acceptor pairs for FRET and 2) to use this information to improve the performance of our Epac-based cAMP FRET sensor as a model for single-polypeptide type FRET sensors in general. Performances of linker constructs and cAMP sensors were evaluated bearing in mind the different requisites for FLIM and ratiometric detection. Based on our results, we recommend CFP^nd^-Epac-cp^173^Venus and CFP^nd^Δ-Epac-cp^173^Venus-Venus (which is somewhat larger, but completely devoid of speckles) as best choices for ratiometric as well as quantitative SE detection. Both show a 44% increase to the already robust FRET span of the originally published sensor [1] and in addition are much more refractory to pH changes and UV illumination. For detection at longer wavelengths and in particular for FLIM measurements, we present GFP^nd^Δ-Epac-mRFP and GFP^nd^-Epac-mCherry as preferred choices. For sensitized emission experiments, the latter outperforms the former in having better acceptor photostability at the expense of a somewhat decreased FRET span.

Noting that a wide, but by no means exhaustive range of FPs was included in this study, our further observations should be helpful in guiding future development of FRET sensors. These observations may be summarized as follows.

First, within the linker construct the majority of FP combinations yielded decent to excellent FRET efficiency (0.1<E<0.31). Spectral match of donors to acceptors varies widely between these constructs and therefore it appears that the amount of spectral overlap is not the prime determinant of FRET efficiency in these constructs. In general, good FRET of a particular FP pair in the linker construct correlated with a good FRET span in the Epac sensor, which should come as no surprise for a sensor that relies on cAMP-induced loss of basal FRET efficiency.

Second, by comparing results from the linker constructs, the general trend is that C-terminal truncation of the donor (the CFPΔ/GFPΔ mutants) has a beneficial effect on FRET efficiency, particularly when introduced in constructs exhibiting moderate basal E values. This improvement is seen irrespective of the acceptor species, but the similar truncation did not result in significant improvement in the Epac sensors, most likely reflecting a different orientation and/or degree of rotational freedom in the latter constructs.

Third, when introduced in constructs with moderate FRET levels, double-acceptor moieties also significantly enhanced E values (compare CFP-linker-Venus to CFP-linker-Venus-Venus and CFP-linker-cp^173^Venus-cp^173^Venus; compare CFP-linker-mCherry to CFP-linker-mCherry-mCherry; compare CFP-linker-HcRed to CFP-linker-HcRed-HcRed). Again, this effect was not seen in constructs that already showed high E values (compare CFPΔ-linker-Venus to CFPΔ-linker-Venus-Venus and CFPΔ-linker-cp^173^Venus-Venus; compare CFP-linker-cp^173^Venus to CFP-linker-cp^173^Venus-cp^173^Venus). In the cAMP sensor, no beneficial effects were observed (Figure 3). However, irrespective of the effects of this modification on E values, it is worthwhile to include the tandem acceptors in the FP repertoire because it cured the tendency to form aggregates displayed by some constructs and can substitute for extensive testing of circularly permuted variants.

Fourth, the range of CFP-redFP constructs demonstrates that various red FPs are good acceptors for CFP. These constructs were prepared in order to boost FRET span by having better spectral separation between donors and acceptors, but in our Epac sensor this theoretical advantage did not materialize.

Fifth, we observed clear positive effects of incorporation of circular permutation mutants of Venus in the linker constructs and Epac sensors. This would seem to indicate that dipole alignment is all-important, but putting our observations in the context of published data may raise some doubt on this interpretation. Both in the linker- and the Epac constructs substitution of Venus by cp^173^Venus yielded the largest improvement. This would indicate that in both these constructs N- and C-terminus of the inserts are in comparable locations and that rotational freedom of the FPs is similar. This is unlikely, given the nature of the very different inserts. Even more remarkable is that cp^173^ also is the favorite acceptor in other single chain FRET sensors published to date [27] [37] [38]. Thus, the widespread preference for cp^173^Venus acceptors raises the suspicion that other qualities, rather than the dipole orientation, cause cp^173^Venus to be such a good acceptor.

Throughout this study we have emphasized that besides FRET efficiency or -span other qualities are equally important for FRET sensors. Slow green-to-red maturation terminated some of the otherwise most promising FRET pairs, e.g. those with tdTomato as acceptor. Similarly, the tendency to form bright speckles (aggregates; vesicles) is an undesirable property that was found in some constructs. We found that introduction of non-dimerizing mutations (A206K) in the FPs had little effect on speckle formation. However, the insert was of significant influence, as the Epac constructs generally were less hampered by speckles. Orientation of donors and acceptors within the linker construct conceivably is such that it allows both FPs of one polypeptide to interact with those of another, effectively increasing the avidity. Importantly, introduction of tandem accepters cured this flaw. We speculate that this is due to internal dimerization of the tandem acceptors but this was not investigated systematically.

As the double exponential fluorescence decay of CFP is inherently poorly suited for FLIM we based our FLIM sensors on GFP. However, the red acceptors for GFP all exhibit some degree of green-to-red maturation. In practice, mRFP and mCherry which mature relatively fast (<1 hr and <15 min, respectively) performed well in FLIM sensors. For future development, one must also consider another promising approach that was recently reported. Here, the donor is combined with a dark (i.e., non-fluorescent) acceptor termed REACh, for Resonance Energy Accepting Chromoprotein [13]. REACh is a YFP variant that may be combined with GFP in the Epac sensors, allowing lifetime measurements of this single-exponential donor and obviating the need for narrow spectral filtering of GFP emission.

## Methods

### Constructs

The previously published construct CFP^nd^-Epac1(ΔDEP, CD)-YFP^nd^ [1] was used to generate all described constructs. Linker constructs were obtained by excising the Epac1(ΔDEP, CD) domain using EcoRV/NheI and replacing it with a linker (GATATC CTTTTGCTAGC , restriction sites EcoRV-NheI are underlined), yielding a 13aa linker (SGLRSRYPFASEL) between the two fluorophores. CFP^d^ and YFP^d^ were obtained by introducing the pointmutation K206A [19] in CFP^nd^ and YFP^nd^ using QuikChange (Stratagene). Donor fluorophore truncations (GFPΔ/CFPΔ) were generated by PCR and lacked the C-terminal part of the fluorophore (GITLGMDELYK) as well as the N-terminal part of the linker (SGLRS). Using HindIII/EcoRV, these PCR products were used to replace CFP. Venus and cpVenus FPs were generated by PCR using the following templates: Venus [21] and cpVenus (from Ycam 3.2, 3.3, 3.6, 3.7 and 3.9; [27]). Using NheI/EcoRI these PCR products were used to replace YFP. Tandem versions were generated by digesting EcoRI/Xba and inserting an additional linker (AATTTTGTCTTCCTGATCGGCGCCGCAGGAATACTCTTCGTATCTAGAGTGAATTCCTAA, newly situated restriction sites Xba-EcoRI are underlined). This linker was digested with XbaI/EcoRI and an additional fluorophore was inserted using NheI/EcoRI, resulting in two acceptor fluorophores separated by a 19aa (PNFVFLIGAAGILFVSGEL) linker. For RFP replacements an identical strategy was followed, however, using additional templates mRFP1 [28], mCherry, tdTomato, mOrange [8], HcRed [34] and tdHcRed [35].

### Materials

IBMX, Forskolin and Ionomycin were obtained from Calbiochem-Novabiochem Corp. (La Jolla, CA).

### Cell culture and transfection

HEK293 cells were seeded on 25-mm glass coverslips in six-well plates in DMEM supplemented with 10% FCS and antibiotics. Constructs were transfected using calcium phosphate precipitate, at ∼0.8 µg DNA per well.

### Microscopy

Cells in bicarbonate-buffered saline (containing, in mM, 140 NaCl, 5 KCl, 1 MgCl2, 1 CaCl2, 10 glucose, 23 NaHCO3, 10 HEPES), pH 7.2, kept under 5% CO2, at 37°C were routinely inspected using a Leica DM-6000 inverted microscope with 63x, 1.32 NA oil immersion objective. For the experiments described in Figure 7A, a triple band fluorescence filter cube (CFP-YFP-RFP), type X154 (Omega) was used. Confocal images were collected using a TCS-SP5 confocal scanhead attached to the DM6000 microscope (Leica, Mannheim, Germany). Agonists and inhibitors were added from concentrated stocks.

For spectral fingerprinting, an Ocean Optics Type USB2000 spectrometer (Dunedin, Florida, USA) was fitted to an inverted microscope. The emission of single transfected cells was captured (integration time, 1–8 s). Excitation was from a arc lamp equipped with a monochromator.

To assess UV sensitivity (Figure 6), cells were followed using time-lapse imaging. After establishing a baseline, cells were subjected to a brief (1 s = 1,000,000 µs) flash of UV illumination using a DAPI filter cube and an EL-6000 light source (Leica) at full power. For lower exposures, the exposure was attenuated by different combinations of shutter times and neutral density filters, and expressed as equivalent shutter time in µs. Flash-induced change in YFP/CFP ratio was plotted versus equivalent exposure time.

### Dynamic FRET monitoring

Cells on coverslips were placed on an inverted NIKON Diaphot microscope and excited at 425 nm (CFP) or 470 nm (GFP). Emission was detected using optical filters as follows: (band-pass filters) CFP, 470±20 nm; GFP, 510±30 nm; YFP, 530±25 nm; (long pass filter) orange and red FP variants, 590 LP filter. Data from donor and acceptor were collected simultaneously, digitized and FRET was expressed as ratio of donor to acceptor signals. The FRET value of the baseline was set to 1.0 at the onset of the experiments. Cells were then stimulated with 25 µM Forskolin and 100 µM IBMX to maximally raise the intracellular cAMP concentration. Changes are expressed as percent deviation from the initial value of 1.0. Data are from 6–20 cells per experiment. Note that for FRET pairs with widely different spectral properties the FRET span can not be quantitatively compared because of inevitable differences in filters and detector sensitivity.

### Fluorescence lifetime imaging

FLIM experiments were performed on an inverted Leica DM-IRE2 microscope using a Lambert Instruments (Leutingewolde, the Netherlands) frequency domain lifetime attachment, controlled by the vendors LI-FLIM software. CFP & GFP were excited with ∼4 mW of light from a 442 nm and a 470 nm LED, respectively, modulated at 40 MHz and emission was collected at 450–490 and 500–540 nm, respectively, using an intensified CCD camera. Lifetimes were referenced to a 1 µM solution of Rhodamine-G6 in saline that was set at 4.11 ns lifetime. The measured lifetimes (calculated from phase differences) of CFP and GFP in the absence of acceptors were 2.7 ns. FRET efficiency E was calculated as E = 1−(measured lifetime of FRET pair)/(measured lifetime of donor). FRET efficiencies are means of 20–400 cells per construct.
